# Sociomateriality in Action: Theorizing Change in Sociomaterial Practices of Working from Home

**DOI:** 10.1007/s12599-023-00796-w

**Published:** 2023-04-04

**Authors:** Lena Waizenegger, Kai Schaedlich, Bill Doolin

**Affiliations:** Auckland University of Technology, Auckland, 1010 New Zealand

**Keywords:** COVID-19 pandemic, Home office, Sociomaterial practices, Sensemaking, Working from home

## Abstract

The COVID-19 pandemic has led to an enforced ‘big bang’ adoption of working from home, involving the rapid implementation and diffusion of digital collaboration technologies. This radical shift to enforced working from home led to substantial changes in the practice of work. Using a qualitative research approach and drawing on the interview accounts of 29 knowledge workers required to work from home during the pandemic, the study identified five sociomaterial practices that were significantly disrupted and required reconfiguration of their constitutive social and material elements to renew them. The paper further shows evidence of the ongoing evolution of those sociomaterial practices among the participants, as temporary breakdowns in their performance led to further adjustments and fine-tuning. The study extends the body of knowledge on working from home and provides a fine-grained analysis of specific complexities of sociomaterial practice and change as actors utilize conceptual and contextual sensemaking to perceive and exploit possibilities for action in their unfolding practice of work. Against the backdrop of the increasing adoption of hybrid working in the aftermath of the pandemic, the paper offers four pillars derived from the findings that support the establishment of a conducive working from home environment.

## Introduction

For all that we’ve been able to achieve while many of us have been separated, the truth is that there has been something essential missing from this past year: each other. (Tim Cook, as cited in Delbert 2021)

The COVID-19 pandemic and the adaptations to organizational and personal life that it required have had a profound impact on business, organizations, culture, and society (O’Leary 2020). In late March 2020, it was estimated that more than 3.4 billion people in 84 countries were confined to their homes, resulting in millions of employees becoming remote workers ‘overnight’ (Bouziri et al. 2020). Many organizations worldwide had to rapidly adopt or diffuse digital technologies to facilitate communication and collaboration in their emergency remote workforce. In particular, the radical shift to enforced working from home during the pandemic (Waizenegger et al. 2020) triggered the wide-scale adoption of digital technology-driven or technologically intensive work practices, the integration and adaptation of which are not yet well understood (Carroll and Conboy 2020; Klein and Watson-Manheim 2021; Nagel 2020; Tønnessen et al. 2021). As we move on from the COVID-19 pandemic, it is likely that these new practices will remain important as part of a ‘new normal’ of work (Carroll and Conboy 2020; Oksanen et al. 2021; O’Leary 2020).

The adoption of flexible or permanent remote work is underlined by the announcements of several big tech companies such as Airbnb (Goldberg 2022), Slack (Stoller 2021), and Shopify (Cheng 2020), which have allowed their employees to continue working remotely, even after the pandemic. One of the factors that influence the decision to adopt a permanently flexible or remote workforce is the positive perception about the switch from office work to remote work. A study based on 1200 US workers and 133 US executives revealed that 71% of employees and 83% of employers perceived the shift to remote working as successful (PWC 2021). Other studies have highlighted the perceived benefits of ‘hybrid working’, combining periods of remote working with a partial return to the office, for some employees and organizations (Barrero et al. 2021; Office for National Statistics 2022; Smite et al. 2022).

Given the possibility of new variants of the COVID-19 virus and waves of repeated infections, as well as the potential large-scale adoption of hybrid working in the longer term, it is important to understand how employees navigated enforced working from home to pursue their work commitments while safeguarding their well-being. Existing research on enforced working from home is different from that on pre-COVID-19 remote working or teleworking (Vartiainen 2021) and shows a very diverse picture of the effects of enforced working from home on employees’ work outcomes and well-being. While existing studies single out specific challenges and explore individual work outcomes such as productivity levels (Ajzen and Taskin 2021) or knowledge sharing and innovation capabilities (Tønnessen et al. 2021), an understanding of how the practice of work changes under enforced working from home is missing. Insights on how employees make sense of and adapt to enforced working from home using digital technologies would not only provide fertile ground for theorizing this phenomenon but also assist organizations and employees in mitigating the challenges that arise in providing remote or hybrid working conditions.

To attend to this gap, we combine the theoretical lenses of sociomaterial practice (Orlikowski 2007, 2010) and sensemaking within a practice world (Sandberg and Tsoukas 2015, 2020) to pose the research question: *How do employees navigate enforced working from home and how do their sociomaterial practices of work change as a consequence?*

By focusing on practice, we are interested in how individuals accomplish workplace activities in the particular situations and circumstances (Luff et al. 2000) of enforced working from home, including how digital technologies are appropriated and used in these contexts. This directs our attention to the situated management and orchestration of multiple devices and applications and, in particular, “how orchestrating them becomes a central aspect of the very … practices they facilitate” (Rossitto et al. 2014, p. 141). Taking a sociomaterial perspective on practice shifts our analytical attention from separate and distinct social and material entities with preassigned meanings and properties to a performative view of always already existing sociomaterial entanglements enacted in everyday organizational practices (Holeman 2018; Orlikowski 2007,2010; Suchman 2007). From such a perspective, the materiality of digital technologies, but also bodies and physical spaces, and their constitution of work practices are implicated within deeply entangled sociomaterial assemblages (Orlikowski 2007; Suchman 2007).

Embedded in the practices they are enacting and entangled with other actors and material objects, actors’ practical engagement with their organizational and work activities is informed by sensemaking (Sandberg and Tsoukas 2011, 2020). Engaging in practice means engaging with the situation at hand – the unfolding activity – a process in which sensemaking is deeply implicated: “sensemaking involves turning circumstances into a situation that is comprehended … and that serves as a springboard into action” (Weick et al. 2005, p. 409).

We address our research question using the findings from a qualitative research study (Sarker et al. 2018) involving semi-structured interviews with knowledge workers who were required to work from home during the COVID-19 pandemic in 2020. We ‘zoom in’ on why and how work practices changed (Nicolini 2009) as a result of the significant disruption caused by enforced working from home. In particular, we focus on five sociomaterial practices that emerged as workers made sense of the disruption to their everyday routines and reconfigured and fine-tuned the sociomaterial enactment of work in the context of working from home during the pandemic: scheduled online meetings, ad-hoc communication, online socializing, managing work space and time, and accessing organizational resources. Our study explores how individuals engaged with digital technologies and adapted to evolving situations in working from home environments from within the sociomaterial practices in which they were positioned.

## Theoretical Background

### Working from Home During the COVID-19 Pandemic

While the COVID-19 pandemic led to rapid mass adoption of working from home, the ‘spatial revolution’ (Felstead and Henseke 2017), which is the liberation from the traditional ‘work in the office’ to alternative forms of working such as remote working or telework (Vartiainen 2021), had already begun prior to the pandemic. This trend was enabled through the expansion of high-speed internet and the adoption and diffusion of digital technologies (Felstead and Henseke 2017; Grant et al. 2013; Song and Gao 2020). Organizations offered remote or teleworking options to enhance employees’ work performance, increase their organizational commitment, cut costs – in particular operational costs such as office space and employees’ overtime – and allow employees to better manage their private and work commitments, affording an improved work-life balance (Ajzen and Taskin 2021). Many employees embraced these alternative ways of working because of the perceived benefits associated with them, such as improved job satisfaction, improved work-life balance, enhanced job effectiveness, and increased autonomy (Felstead and Henseke 2017; Grant et al. 2013).

Despite the spatial revolution already unfolding, the outbreak of COVID-19 and the resulting governmental stay-at-home orders in early 2020 resulted in an unprecedented ‘big bang’ adoption of enforced working from home (Caldeira et al. 2022; Carroll and Conboy 2020). However, in contrast to working from home before the pandemic, many people now had no choice, very little time to prepare, and often no allocated place to work in their private homes. Further, work had to continue alongside a range of other impacts, including financial pressures, feelings of fear and anxiety, and the need to fulfill multiple roles simultaneously (e.g., a mother, a consultant, a wife), with school closures creating additional childcare work for parents and sometimes leading to increased stress levels and exhaustion (Waizenegger et al. 2020).

Similar to the literature on remote work and teleworking prior to the pandemic (Bailey and Kurland 2002; Nakrošienė et al. 2019), research on the effects of enforced working from home on work outcomes and employees’ well-being does not show a uniform picture. While some studies showcase the benefits of enforced working from home (Ajzen and Taskin 2021; De-la-Calle-Durán and Rodríguez-Sánchez 2021; Tønnessen et al. 2021), others highlight the challenges (Nadler 2020; Oksanen et al. 2021; Savolainen et al. 2021), and yet others show no effects at all (Awada et al. 2021; Mattern et al. 2021). The experiences of individuals in enforced working from home settings are subtle and often differ depending on various demographic factors such as gender, age, professional background (Awada et al. 2021; Savolainen et al. 2021), and experience with digital technologies and social media (Oksanen et al. 2021).

Despite the radical shift to enforced working from home, some people benefited from the new work model and experienced various benefits. For example, some executives perceived online meetings as more productive than in-person meetings, as participants were more focused on the business matters at hand, showing the potential of digital collaboration platforms to enhance work efficiency (Richter 2020). Research found that working from home can lead to increased energy levels among workers, enhancing job involvement and productivity levels (De-la-Calle-Durán and Rodríguez-Sánchez 2021). Similar results were observed in a study conducted at an insurance company in Brussels. The improvement in productivity levels was mainly achieved through a reduction in interruptions from colleagues that usually occur while working in the organizational office (Ajzen and Taskin 2021). Further, enforced working from home also had an impact on the way employees shared their knowledge and innovated, which is now mainly facilitated through digital technologies. A study based on 237 Norwegian knowledge workers found that 42% of their participants increased their digital knowledge sharing within the organization, while 29% indicated a decrease. Further, both internal and external knowledge sharing positively impacted the employees’ performance in creating novel ideas, products and services, and procedures that might add value to the organization (Tønnessen et al. 2021).

Other studies claim that enforced working from home has little or no effect on employees’ work outcomes and well-being. For example, Awada et al. (2021) found that while the number of hours spent at the workstation at home increased by approximately 1.5 h, the perceived productivity of workers comparing pre-COVID-19 in-office work to enforced working from home did not change. Another study, analyzing diary entries of 37 participants over a four-week period at a German university, found that enforced working from home neither led to an inability to detach from work nor communication overload and that employees’ stress levels did not increase (Mattern et al. 2021).

A further group of studies identified a variety of challenges that employers and employees struggled with that often led to detrimental effects on work outcomes and well-being. In order to still be able to collaborate and communicate in the new emergency remote working model, organizations had to pivot and implement or diffuse digital technologies and social media applications rapidly (Oksanen et al. 2021). This digital leap led to the accelerated and large-scale adoption of various enterprise social media (e.g., Microsoft Teams, Workplace by Facebook, Yammer) and a stark increase in formal social media communication among employees. Employees who did not use social media for work purposes before the pandemic often felt overwhelmed and experienced a high level of technostress and work exhaustion (Oksanen et al. 2021). Some employees thought they were increasingly surveilled by managers and colleagues and therefore checked incoming emails more frequently to prove their engagement at work, in turn leading to increased stress levels (Richter 2020). Companies relied heavily on video conferencing platforms like Zoom and Google Hangouts to shift their scheduled and ad-hoc meetings online. This is reflected in the number of daily Zoom meeting participants, which skyrocketed from 10 million in December 2019 to 300 million in April 2020 (Evans 2020). The only way people could discuss issues, distribute tasks, brainstorm new ideas, or transfer knowledge was through digital channels and, in particular, video conferencing tools. Many employees spent the majority of their workday in online meetings leading to the so-called “Zoom fatigue” (Nadler 2020, p. 1).

Although online meetings were often the only option to achieve collaboration goals and maintain contact with clients, many employees still preferred in-person meetings as they were perceived as more suitable for knowledge sharing. Further, missing the familiarity of their organizational office and their colleagues, employees often felt isolated and detached from the wider team, causing enhanced stress levels (Ajzen and Taskin 2021). These effects could be aggravated for those employees that usually collaborated closely and who had no experience with working from home, potentially affecting their mental health and decreasing productivity levels (Koren and Pető 2020). Lastly, many employees suffered from COVID-19 anxiety induced by the uncertain consequences of the pandemic, including concerns about their job security (De-la-Calle-Durán and Rodríguez-Sánchez 2021; Savolainen et al. 2021). These uncertainties had an enhanced impact on workers who suffered from loneliness and emotional stress and ranked high on neuroticism. Further, research shows that women and younger workers experienced higher COVID-19-induced anxiety levels (Savolainen et al. 2021).

While existing research studies identify and explore the various challenges that employers and employees face in this ‘new normal’ (Carroll and Conboy 2020), accounts that analyze the sociomaterial complexities of enforced working from home and explain how work practices changed in response to these challenges are missing. Such an account focuses on the practical sociomaterial specifics of working from this changed context as something that individuals do in their ongoing activities and actions (Holeman 2018; Styhre 2011). The aim is to explain how workers navigate their changed circumstances, make sense of the disruptions they face, and adjust their work practices to pursue their professional commitments and safeguard their well-being while working in their homes (Carroll and Conboy 2020; Tønnessen et al. 2021).

### Sociomaterial Practice

Our study analyzes how employees (individual knowledge workers) performed work activities in the private space of their homes during the COVID-19 pandemic using various digital technologies. Underlying our analysis is an understanding of work as a sociomaterial practice. A practice perspective focuses attention on situated activities and their performance. It recognizes that social or organizational life is an ongoing production that emerges through people’s everyday actions. Practices are not just what people do but rather how the doing of those things is productive of coherence and meaning and continually enacts a phenomenon over time (Feldman and Orlikowski 2011; Leonardi 2015; Nicolini 2012). From a practice lens, work is thus “an ongoing routinized and recurrent accomplishment” (Nicolini 2012, p. 3). We use the concept of *sociomaterial practice* to acknowledge the importance and inherence of materiality, both physical and digital, in the production of social life (Feldman and Orlikowski 2011; Holeman 2018): “*every* organizational practice is *always* bound with materiality … The social and the material are *constitutively entangled* in everyday life” (Orlikowski 2007, p. 1436–1437, italics in original).

Contemporary knowledge workers already inhabit a work environment saturated with digital tools and communication media (Wajcman and Rose 2011). This ubiquity has made digital technology an inherent part of the everyday flow of activities in organizations (Gerson et al. 2008). However, as noted earlier, the enforced working from home induced by the COVID-19 pandemic intensified the centrality of and reliance on technology-driven work practices. Taking a sociomaterial practice perspective allows us to explore how such work is inextricably bound up with the use of digital technologies and tools (Bjørn and Østerlund 2014; Holeman 2018) and how it unfolds “as a form of constitutive entanglement of social and material resources at hand” (Styhre 2011, p. 384). In other words, viewing work practices as sociomaterial allows us to focus on how they are enacted or brought into being in emergent human-technology configurations (Bjørn and Østerlund 2014; Klein and Watson-Manheim 2021; Orlikowski 2007; Schultze et al. 2020; Suchman 2012). Rather than treating the social and material elements of these configurations as discrete analytical categories, sociomateriality emphasizes how they are constitutively entangled and enacted together in the performed relations of work practices (Orlikowski 2007, 2010). Seen in this way, agency or the capacity for action arises from the relational whole of sociomaterial practice (Sandberg and Tsoukas 2011) rather than from discrete and separate social actors and material artifacts (Bjørn and Boulus-Rødje 2015; Suchman 2007).

To operationalize our analysis of how work practices changed under enforced working from home, we draw on a conceptualization of sociomaterial practice as emergent in the situated flow of everyday action (Hultin et al. 2021; Mousavi Baygi et al. 2021). From this perspective, change occurs contingently from within practice rather than at the external direction of originating human actors. The actors are there, inhabiting the practice world, but change is continuously emerging in the unfolding flow of practice (Mousavi Baygi et al. 2021). Work is not just intentionally performed but also ‘attentionally undergone’ as actors respond to the flow of practice (Hultin et al. 2021) by being attentive to possibilities for action that potentially shape the ongoing flow (Klein and Watson-Manheim 2021). Immersed in the temporal flow of practice, they experience a sense of “the practical necessities, uncertainties, and urgencies in which practitioners are typically entangled” (Sandberg and Tsoukas 2011, p. 342). Further, the flow of practices conditions both human subjects and material objects to become enacted and act in particular ways (Hultin et al. 2021).

More specifically, as skillful practitioners, workers engage with digital technologies and other material artifacts in their work routine and ongoing practice (Bjørn and Østerlund 2014; Sandberg and Tsoukas 2020). Their use of tools and technologies becomes a taken for granted part of the sociomaterial configuration that is work performed in practice: “In our normal everyday coping, we deal with ready-to-hand equipment without any thought at all” (Dreyfus 1997, as cited in Bjørn and Østerlund 2014, p. 16). As Sandberg and Tsoukas (2020) put it, “the world no longer appears as a collection of contingently linked items but as a meaningful relational whole that affords certain possibilities for action” (p. 5).

Sandberg and Tsoukas (2011) refer to this as ‘absorbed coping’, a mode of engagement with the world in which actors respond spontaneously to the unfolding situation without a deliberate focus on the sociomaterial practice itself. This is because, under normal circumstances, organizational actors are immersed in the temporal flow of a sociomaterial practice such as work without being consciously aware of their involvement in it. They are utilizing ‘immanent’ sensemaking that is practical and directed at enacting routine activities. In performing the ongoing actions of their practice, actors are already familiar with the meaning of a situation and know in a practical sense what must be done (Sandberg and Tsoukas 2020). This is a process of constant tuning and instant adjustments to practice as it unfolds: “sense and action are not separate but merged as a single ongoing response to the particularities of the unfolding situation” (Sandberg and Tsoukas 2020, p. 10).

More extrinsic sensemaking is triggered by ‘disruptive ambiguity’ (Weick et al. 2005), a puzzling or equivocal event that disturbs or interrupts an actor’s ongoing activity, obliging them to work to understand the disruption and make it sensible (Maitlis and Christianson 2014; Weick 1995). This involves noticing cues that indicate variance from the ‘normal’ routine activity and bracketing them – labeling them in ways that impose a simplified order on the world: “render[ing] the subjective into something more tangible” (Weick 1995, p. 14). These extracted cues are interpreted to develop a more complete sense of the interrupted activity, and then this interpretation is acted on to explore whether it repairs and restores or renews the interrupted activity (Sandberg and Tsoukas 2015; Weick et al. 2005).

When something occurs in the performance of practice that interrupts an actor’s absorbed coping, the actor begins to focus deliberately on the practice and its constitutive social and material elements as discrete from the relational whole (Sandberg and Tsoukas 2011). Such disruptions or ‘breakdowns’ in actors’ absorbed coping can occur through thwarted expectations if the consequences of their practices are unintended or differ from existing performance standards. Alternatively, actors may experience the emergence of deviations from established routines or become aware of possible different practices, each of which can disrupt their absorbed coping (Sandberg and Tsoukas 2011). Mousavi Baygi et al. (2021) suggest that these moments of breakdown in the current flow of action occur when alternative flows of action contingently intersect or become confluent. Such correspondences in flows of action can be both generative and disruptive, leading to an episode of more deliberate sensemaking. Actors engaged in the practice of work sense at opportune moments the emergence of new possibilities for action in different sociomaterial configurations as these are performed in practice (Mousavi Baygi et al. 2021).

Focusing on moments of breakdown in the flow of practice enables us to investigate how important aspects of the enacted relations that constitute the ‘wholeness’ of a sociomaterial practice become salient, visible, and available to the actor (Bjørn and Boulus-Rødje 2015). When an actor’s performance of a work activity does not occur as anticipated, the actor starts reflecting on it, paying specific attention to restoring the activity at hand and continuing their work (Dowling et al. 2018; Mazmanian et al. 2014; Sandberg and Tsoukas 2011). The temporary breakdown in absorbed coping momentarily brings into view the relational whole of a sociomaterial practice, as the actor focuses deliberate attention on what has become problematic in that practice while remaining involved and engaged in the practical activity. The actor engages in ‘involved-deliberate’ sensemaking in order to continue working on their tasks (Sandberg and Tsoukas 2011). This is contextual sensemaking – not just being aware of the circumstances that caused the interruption but attentionally making sense of the affordances offered or possibilities available in the unfolding situation in order to find an appropriate way to restore the interrupted activity by adjusting elements of their sociomaterial practice from within that practice (Sandberg and Tsoukas 2011). Possibilities for action that arise are sensed by an actor by being attentionally attuned to the flow of action in their engagement with the practice (Mousavi Baygi et al. 2021). Attentionality requires responsiveness and activity to actualize those possibilities for action: “ongoing attentional action [is required] to cope with mundane contingencies and coincidences” (Hultin et al. 2021, p. 612).

The rapid transition to enforced working from home during the COVID-19 pandemic caused a sudden disruption to work routines, typically replacing in-person work processes with dispersed work (Caldeira et al. 2022). This represents a more complete breakdown in an actor’s absorbed coping than the temporary breakdowns described above. In a complete breakdown, the disturbance or interruption is so significant or persistent that the actor becomes detached from the practical activity at hand and unable to act in the previous, routine way: “what agents have taken for granted, which has enabled them to engage immanently with their tasks, now breaks down” (Sandberg and Tsoukas 2020, p. 13). The disruption of established meanings creates a separation between sense and action, foregrounding the array of social and material elements that previously constituted the relational whole of sociomaterial practice. No longer immersed in the routine flow of their work, the actor shifts to engaging with their activity in a more abstract way, reviewing the properties of the situation and making conceptual sense of the problem. They utilize ‘detached-deliberate’ sensemaking to focus their attention on establishing the likely causes of the breakdown and how they may be addressed to solve the problem. The outcome of this conceptual sensemaking process is a reconfigured or new entanglement of sociomaterial practice that renews how the problematic activity is performed (Sandberg and Tsoukas 2011, 2020).

**Fig. 1:**
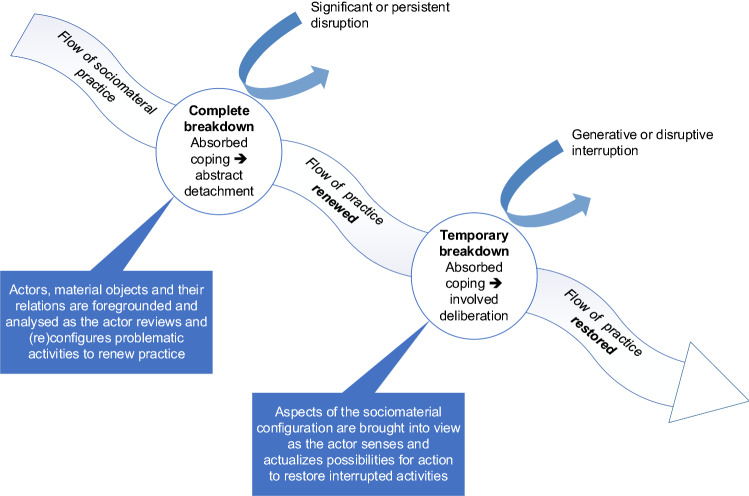
The flow of sociomaterial practice

Figure 1 is a diagrammatic representation of our conceptualization of the flow of sociomaterial practice and how it evolves as changes in the situation of work, for example, unfold. As they work, organizational actors are immersed in the ongoing flow of their practice, routinely performing their tasks and activities. Their agency as workers is entangled with the materiality of the tools and technologies they use and the social or organizational context in which they are used (Wajcman and Rose 2011). Of course, the flow of practice does not always continue on smoothly. The actor’s absorbed coping in routine practice is disturbed and breaks down to varying extents as the unfolding situation interrupts the flow or introduces different possible flows of action (Mousavi Baygi et al. 2021). Most of the time, these disruptions represent temporary breakdowns that trigger involved deliberation by the actor as they adjust elements of the sociomaterial configuration that offer possibilities for repairing and restoring the interrupted activity. At other times, the actor may experience a disruptive incident that causes a more complete breakdown in the routine accomplishment of practice. In such cases, practice cannot continue unchanged and must be (re)configured and renewed.

Our depiction of the flow of practice in Fig. 1 is an idealized one, although it broadly follows what we observed in our study: a complete breakdown caused by the disruption to office-based work during the COVID-19 pandemic that resulted in a renewal of the sociomaterial practice of work in a new setting, followed by subsequent temporary breakdowns as actors progressively adjusted their routines in working from home. Other sequences are of course possible, including where consecutive or coinciding temporary breakdowns cumulatively lead to shifts in the flow of a practice without the occurrence of a more complete breakdown. Possibilities to act are not conditioned in chronological order by a single preceding event but emerge through actors’ attention to correspondences along multiple flows of action in a sociomaterial practice (Mousavi Baygi et al. 2021).

## Research Design

In order to explore our research question, we use a qualitative research approach guided by the underlying philosophical assumptions of interpretivism (Myers and Walsham 1998; Walsham 2006). In line with our interpretive stance (Myers and Walsham 1998; Neuman 2000), our goal is to (a) understand how employees make sense of the rapid shift to enforced working from home (Waizenegger et al. 2020), the associated challenges, and disruptions to their work; and (b) how this sensemaking results in changes to their sociomaterial practice of work.

### Data Collection

Our data set is drawn from a study of the experiences of knowledge workers that were forced to work from home due to the COVID-19 restrictions imposed in many countries during 2020. A team of four interviewers, including the lead author of this paper, conducted 29 qualitative interviews following the dramaturgical model proposed by Myers and Newman (2007). In this approach, the interview is treated as a social interaction in which the interviewer also plays a role, not only gathering data but also actively constructing knowledge (Fontana and Frey 2000). This meant that the interviewers took a reflexive approach to be sensitive to how their own experiences of working from home during the pandemic may have shaped the interview process.

The interviews took place in April 2020. As the interviewer and interviewee were often based in different cities or countries, and the COVID-19 lockdown restrictions in many countries did not allow in-person interviews, the interviews were conducted virtually using one of two popular audiovisual digital communication platforms. The participants had diverse demographic backgrounds, including different countries of residence, age cohorts, gender, professional roles, parental and marital status, and working from home experience, as presented in Table 1. While 66% of our participants were based in New Zealand during the time of data collection, the remaining 34% were located in Europe, North America, and Australia. Their ages ranged from 20 to 50, with the median age range being 30–40 years. Their occupations were in a diverse range of industries, including HR and recruitment, banking and finance, information technology, construction and engineering, consulting and project management, consumer goods, and academia. Most participants (79%) lived with a partner, and just over a quarter (28%) had children living with them. Most of our participants worked in an organizational office prior to COVID-19, and just under half (48%) had some prior experience working from home on an ad-hoc basis prior to the pandemic.

**Table 1:** Demographic details of participants

Pseudonym	Country	Age	Gender	Role	Has children	Has partner	Experience WFH pre-pandemic
Anna	New Zealand	20–30	Female	Marketing assistant			
Barry	New Zealand	30–40	Male	Chief operating officer		X	
Hamza	New Zealand	30–40	Female	PhD scholar and teaching assistant	X	X	
Andrei	New Zealand	40–50	Male	Project management coach		X	X
Oliver	Germany	30–40	Male	Senior marketing manager	X	X	X
Mansour	New Zealand	30–40	Male	Data scientist	X	X	X
Liam	New Zealand	30–40	Male	Civil engineering technician			
Liz	New Zealand	30–40	Female	Marketing assistant	X	X	X
Rajani	New Zealand	20–30	Female	Web developer			
Salim	New Zealand	30–40	Male	Lecturer			
Adam	Sweden	30–40	Male	Program manager		X	X
Wendy	New Zealand	30–40	Female	Management consultant		X	
Sia	New Zealand	30–40	Female	Corporate solicitor		X	
Jacques	New Zealand	30–40	Male	Business coach	X	X	X
Nick	New Zealand	30–40	Male	Program manager	X	X	X
Emilia	Sweden	30–40	Female	Business developer		X	
Jacob	Sweden	20–30	Male	Management consultant		X	
Rachel	New Zealand	20–30	Female	Copy and content writer		X	
Jane	United Kingdom	40–50	Female	Lecturer			X
Harry	New Zealand	30–40	Male	Recruiter		X	
Pete	USA	40–50	Male	Real estate partner		X	X
Laura	Switzerland	40–50	Female	Project and program manager	X	X	X
Garry	New Zealand	30–40	Male	Product manager		X	X
Daniel	New Zealand	40–50	Male	Project management coach		X	
Matt	Germany	30–40	Male	Data analyst			
Alexander	New Zealand	40–50	Male	Account manager		X	
Sandra	United Kingdom	30–40	Female	Report analyst		X	X
Terry	New Zealand	40–50	Female	Management consultant		X	X
Patricia	Australia	40–50	Female	Medical researcher	X	X	X

The interviews were semi-structured and covered three main areas: (a) how lockdown-enforced working from home changed how the participants completed their work tasks; (b) how the use of digital technologies affected their collaboration with others, and (c) how enforced working from home impacted their private lives and work-life balance. The interviews ranged from just over half an hour to one and a half hours, with an average duration of 55 min. The interviews were recorded in all but three cases and afterward transcribed by a professional transcription agency. The interviewers wrote down the key insights from each conversation with a participant immediately after the interview finished.

### Data Analysis

The findings presented in this paper are based on an analysis of the transcribed interviews. A thematic analysis (Braun and Clarke 2006) was conducted by all three authors of the paper. First, we familiarized ourselves with the data by reading the interview transcripts. We then started the coding process using a well-known qualitative data analysis software tool. Our initial codes reflected the patterns that we observed in the data. A codebook was developed that specified each of the codes used. The codebook was regularly discussed among the authors to achieve shared understanding and clarity of the codes, which later facilitated the theorizing process.

After the initial coding process was completed, we developed themes by collating logically coherent codes from the first coding step together. These initial themes focused on work experiences prior to the COVID-19 pandemic, the use of various digital technologies and tools, challenges experienced in working from home, and changes in work and private lives resulting from the shift to working from home. In several iterations, we then reviewed these themes, taking into account our research question, and organized them into a data structure. This took the form of a large table with three columns and comprised a set of higher, second-order themes that we used to group our first-order themes, together with the data excerpts related to them. Having achieved a better understanding of the phenomenon under study and the patterns that emerged from the data, we then identified a suitable theoretical lens that would allow us to explain our findings.

In particular, we used the concepts of sociomaterial practice and sensemaking (Dowling et al. 2018; Hultin and Mähring 2017; Orlikowski 2007; Sandberg and Tsoukas 2011, 2015, 2020) as well as the flow of practice perspective (Hultin et al. 2021; Mousavi Baygi et al. 2021) as theorizing devices that helped us to better understand how our participants navigated the challenges associated with enforced working from home and how, when and why the sociomaterial practices changed due to the drastic shift in the context of work.

We applied an abductive approach (Dubois and Gadde 2002; Mitchell 2018) and went back and forth between the relevant theoretical concepts and our data in order to find the best explanation for our observations in the data. The abductive reasoning enabled us to identify a) the particular sociomaterial practices and activities that broke down due to the abrupt shift to working from home, b) how the participants made sense of those breakdowns, and c) how the sociomaterial practices changed and continuously evolved as a result of their sensemaking and the reconfiguration of the social and material elements constituting the practices.

## Findings

Prior to the radical shift to enforced working from home, our participants engaged in a plethora of different sociomaterial practices at their organizational workplaces, including holding planned or ad-hoc in-person meetings with colleagues and clients, developing reports or presentations, sharing knowledge, training employees, and engaging on online collaboration platforms and public social media. Under normal circumstances, they primarily engaged in these established work practices in a state of absorbed coping, taking for granted the various sociomaterial configurations that underlay their practice and that were ready at hand. The sudden and highly disruptive lockdowns associated with the COVID-19 pandemic typically caused a complete breakdown in this routine work practice.

Organizations and knowledge workers needed to conceptualize and implement new ways of working remotely. This involved interpreting the changed context of action and creating a sense of what was possible by reviewing and reflecting on “the key dimensions of sociomaterial entanglement that constitute each activity” (Gaskin et al. 2014, p. 852) and how they might be enacted differently. While the type of activity undertaken often remained similar, the location of those activities shifted from being co-located in an established workspace to remotely performed from the private space of the workers’ homes. The changed location of work in turn influenced the actor configuration, the tools available for performing work, and even the types of artifacts being worked on. Individual knowledge workers were geographically separated and physically isolated, often lacking access to physical and specialized infrastructure, and reliant on the modality and capacities of digital technologies for communication and collaboration with their co-workers (Gaskin et al. 2014).

The novel ways that workers experienced, interpreted, and navigated enforced working from home shaped and reconfigured work practices as workers interacted with the physical setting of home-based workspaces, the materiality of digital tools and technologies, and the social context in which they were used (Klein and Watson-Manheim 2021; Wajcman and Rose 2011). Workers needed to develop a contextual sense of the new or reconfigured activities in order to perform them in practice. However, when work and private life take place in the same environment, a different set of interruptions to practice are introduced (Caldeira et al. 2022), leading to ongoing temporary breakdowns in the flow of action. This requires continuous adjustment of workers’ practices through experimentation and fine-tuning (Mousavi Baygi et al. 2021) and a high degree of flexibility in, for example, online communication and collaboration. Eventually, workers will become more fluent in performing the renewed practices, leading to greater stability in the organization and the performance of working from home (Sandberg and Tsoukas 2020).

In the following, we ‘zoom in’ (Nicolini 2009) on five sociomaterial practices that were salient in our analysis of how workers navigated the changed circumstances of work by reconfiguring their work activities and tuning them as their renewed practice unfolded: *scheduled online meetings, ad-hoc communication*, *online socializing*, *managing work space and time*, and *accessing organizational resources*. We devote relatively more space to the first two of these five practices to illustrate our analytical approach. Drawing on our theoretical framework developed above, we explore how these reconfigurations and ongoing adjustments were accomplished by our participants by situating them within the relational whole of sociomaterial practice (Sandberg and Tsoukas 2020). Key to this approach is the understanding that “in agents’ variously deliberate sensemaking, the interrupted activity acquires the status of an object that is amenable to reflection” (Sandberg and Tsoukas 2020, p. 27). This, in turn, enables us, as researchers, to comprehend and explain important aspects of our participants’ unfolding accomplishment of their changed practice (Sandberg and Tsoukas 2020).

### Scheduled Online Meetings

Before the COVID-19-enforced lockdowns, many scheduled meetings were held in person, on site or at a client’s site. People were usually co-located and met as a group, routinely making use of various physical tools and facilities such as meeting rooms, desks, chairs, and whiteboards to discuss ideas, solve problems or develop operational or strategic plans. Enforced working from home clearly disrupted this practice, leading to its complete breakdown as governmental and organizational regulations prevented our participants from meeting in person.

For example, where Andrei previously would travel to clients and conduct his coaching business through in-person meetings, post-lockdown he switched to online meetings using video conferencing services. Forced to detach from his previous practice and engage in deliberate sensemaking to conceptualize the problem, he was able to draw on his prior experience using a particular video calling tool for distributed one-to-one collaboration with an overseas colleague to see the possibility of extending this to working with multiple meeting participants online: *“[Previously] we had in-person meetings and planning days, where the [whiteboard] markers and the stickies and so on showed up. We’ve recently had another full-day session …, but we switched to Zoom for the online collaboration”* (Andrei).

Three points in this example are particularly pertinent. First, Andrei’s eventual use of Zoom for these extended meetings was deliberate – the result of experimentation and fine-tuning his renewed practice of online meetings. His utilization of the initial video calling tool proved unsatisfactory in practice: *“I found [it] to be useful when you have two or three people but as soon as the meeting starts to have more people and they start to be [geographically] dispersed … it starts to break down. Whereas Zoom has a bit more robust capability”* (Andrei). The ‘breakdown’ in the performance of the video calling tool during online meetings disrupted Andrei’s absorbed coping as he engaged in this practice, bringing the tool, previously a taken for granted part of the sociomaterial configuration or relational whole of his practice, into view and focusing his attention on it as problematic and requiring substitution with Zoom to repair that practice.

Second, Andrei relied heavily on visual facilitation in performing his pre-lockdown practice. *“Walking into a presentation room, picking up a marker and … writ[ing] on the whiteboard”* (Andrei) was integral to his work, and whiteboards, whiteboard markers, and sticky notes were clearly valuable artifacts in constituting his sociomaterial practice. In the online environment such physical tools were no longer available. In reconfiguring his meeting practice in an online environment, Andrei instead used a range of digital tools, including a web-based list-making application and an online whiteboard platform. The use of these tools substituted a digital materiality for the physical materiality of the whiteboard he previously used, enabling him to replicate his practice online:

[The online whiteboard] is like a whiteboard should be. It’s a really clever use of technology in that not only can I have simulated stickies and a pen or a pencil and connectors and shapes and so on, I can actually have templates … I can have it in Zoom, share the screen, and the whole team gets to see the whiteboard and I can get them access into it. (Andrei)

Indeed, in some ways, Andrei came to see using the online whiteboard as a superior exercise of sociomaterial agency as he incorporated it into his practice: *“When you put an electronic sticky up and go click, click, click, it’s quite nice and not only that but electronic stickies don’t fall off!”* (Andrei). The third point is that Andrei’s use of virtual conferencing and online collaboration tools to resume his meeting practice post-lockdown was not predetermined or inevitable but emerged as possibilities when other flows of his work practice, in which online collaboration had been experienced, contingently intersected with that he was reviewing and attempting to renew. In this way, the incorporation of digital tools in the reconfigured practice ‘made sense’ as an obvious and appropriate way to perform online meetings. As Andrei commented, *“That’s the natural shift, natural progression towards an electronic format.”*

The development of a renewed sociomaterial practice of online meetings involved increased reliance on and use of the different functionalities of video conferencing platforms and associated digital technology such as an in-built or Web camera, screen-sharing, non-verbal emoticons, and the chat function to accommodate particular actions and activities that were usually executed in an in-person meeting. For example, the use of video functionality was often considered to be crucial in mitigating the loss of visual cues normally present in in-person meetings, which are an important non-verbal communication vehicle to gauge the reactions, moods, and attention of the social actors: “*On Skype, … everyone’s being encouraged to be on camera because you get that connection. You can talk. I can see you, I can see your reactions, I can see your body language, almost as if I’m in a meeting”* (Laura). Mandating camera use was considered to discourage ‘fake listening’, a practice that emerged in online meetings when participants turned off their camera function, effectively hiding behind their screen, and engaging in other activities while being ‘present’ in the online meeting:

I also have a rule that we always have cameras on. My wife works and she doesn’t have to [have the camera on] when they have meetings, which is super annoying, I think, because you don’t see how people feel and people can just do anything while they are just fake listening. (Oliver)

The visual cues provided by video functionality are not always evenly distributed. While the audience can visually follow a presenter, it is more difficult for the latter to keep track of each individual in the gallery of participants in an online meeting. ‘Reading the room’ becomes more difficult, leading to thwarted expectations and a temporary breakdown in the meeting practice as presenters lose their connection with their audience: *“It’s very hard to gauge if people are still paying attention or not without stopping and going, ‘Is everyone listening? Does anyone have questions?’”* (Wendy).

In her work, Wendy participated in the demonstration of new tools. Such scheduled meetings necessarily switched to online meetings post-lockdown: *“It was about twelve people on a training session and where we probably would have got everybody into one meeting room, we just did it over [Microsoft] Teams”* (Wendy). Often such meetings were difficult to keep on time if people asked too many questions. When Wendy experienced such a temporary disruption to the flow of the online meeting, her involved-deliberate or contextual sensemaking suggested possibilities for circumventing these interruptions using the different channels of the video conferencing platform:

Because we only have an hour, we need to stay on track. Something that’s actually nice about the technology is we can say, “Hey, instead of taking yourself off mute and asking your questions, type it in the chat. Then it won’t disrupt the demo, but somebody can still answer your question in real time.” I actually quite like that because it means people can’t hijack your meeting and take you off track and waste time. (Wendy)

In this way, the online meeting practice became adjusted to incorporate ‘multi-channel communication’, with participants using audio and video functionality to participate in the main meeting while the chat function acted as a backchannel to engage in side conversations to clarify questions. This restored practice potentially improved the meeting quality and outcome.

While scheduled meetings could continue in an online space, the actual places people participated from were their own homes. This led to a degree of discomfort for some individuals who resented disclosing aspects of their private lives to their colleagues and tried to keep the two worlds separate. The discomfort they experienced because of this during online meetings in which camera use was expected caused a disruption or temporary breakdown in their practice. The camera function in the online meeting platform became momentarily highlighted and separated out from the relational whole of the practice as they evaluated the functionalities of the platform to find a solution that would restore their comfort and their sociomaterial practice. They started using the blurring function or a virtual background to hide their private environment, maintaining their professional identity and protecting their privacy: *“Simple things like being able to blur your background. We have people who are fiercely private about their own life … and that scares a lot of people, that they’re in their home environment”* (Laura).

Another of our participants felt that a colleague was sharing too much of their home environment in an online meeting to the detriment of their collective professional identity. In the following example, Patricia experienced a deviation from the expected routine of meeting with important stakeholders and asked her colleague to activate the virtual background function as part of his sociomaterial practice in order to restore the desired degree of professionalism:

It looked like a nuclear bunker … I think everyone was taken aback when I said, ‘James, I’m sorry, … either you’re going to need a virtual background or you’re going to need to move your machine because we can’t have your two weeks of food sitting on-screen while we’ve got major donors coming to this event. (Patricia)

### Ad-hoc Communication

Before being forced to work from home due to the pandemic, our participants engaged in various ad-hoc interactions with their colleagues, managers, subordinates, and other internal and external stakeholders, for example, when someone approached them with a specific question or problem in mind or they serendipitously met in the hallway or other areas of the office building. Those in-person ad-hoc encounters were enabled by the office layout and facilities and the associated possibilities for action they offered. For example, the organization’s kitchen or staff lounge allowed physically co-located employees to have a coffee together and share ideas, discuss work problems, or talk about private matters. The radical shift in the actors’ location and configuration after lockdown meant that this sociomaterial practice of ad-hoc communication experienced a complete breakdown, forcing our participants to disengage from the practice and make conceptual sense of the disruption and what might be possible by reviewing and reconfiguring different material and social elements of the practice.

The most common response from our participants was to leverage communication technologies and online collaboration platforms that were available to them, either from other aspects of their prior practice or from an organizational roll-out, enacting these technologies as virtual communication tools in a renewed sociomaterial practice of online ad-hoc communication and coordination. Often multiple communication channels were incorporated into this practice, for example, using one medium to negotiate the availability of a colleague and coordinate an opportunity for consultation (text) before switching to another medium (mobile phone call) to work cooperatively: *“If you want to discuss something quick, not a planned discussion or a scheduled meeting …, I’m using text a lot. I text my colleagues and say, ‘Hey, I want to discuss something, five minutes.’ They say they will call back, which is more efficient”* (Hamza).

In another example, Emilia explains how her use of either her mobile phone or a popular video conferencing tool depends on her audience:

The person that I mostly collaborate with now is my manager – and the group management team members. And those I call, because I know that they’re not using Skype very much, I've tried. But for other colleagues, who I know are using Skype, I would chat with them [by phone] and sometimes we just get on a quick Skype call in the chat. (Emilia)

Note how the particular sociomaterial configuration utilized by Emilia makes sense from within her unfolding practice. She has attempted using video calling with her managerial colleagues (*“I’ve tried”*), but when that proved unsatisfactory, she reverted to phone conversations. On the other hand, less senior colleagues seem more comfortable with video calling, so much so that they and Emilia often spontaneously initiate a video session during a phone call, presumably to leverage the added affordances of video-based communication.

In a further example of how the enactment of particular technologies as tools for ad-hoc communication was shaped by the emergent practice of workers, Nick and his team incorporated email, commonly used for asynchronous communication, as a near-synchronous communication tool for personal conversations in their post-lockdown practice: *“When I’m [working] at home I tend to do a bit more in terms of communication by email, because that’s how everyone else seems to be communicating as well. So, just having a quick chat about stuff”* (Nick). While a particular technology like email is designed with certain functionalities, how it is enacted in the performance of a sociomaterial practice is the outcome of the entwinement of both social and material agency.

As our participants gained experience in using digital technologies to facilitate ad-hoc communication, they made adjustments to their practice to take advantage of the different possibilities they sensed in engaging with particular elements of the sociomaterial configuration constituting that practice. For example, where Liam and his colleagues would previously have visited each other’s offices to discuss an issue or solve a problem while jointly viewing the screen on the office computer monitor, this became impossible under enforced working from home. The result of Liam’s initial sensemaking on circumventing this disruption to his normal work practice was “*to screenshot something and then write a caption about what it is and ask a question or show whatever problem has occurred, and then email it to someone in [another location], if they were the person that’s going to have the answer for you.”* However, Liam came to regard the renewed sociomaterial practice incorporating the asynchronous communication channel offered by email as inefficient compared to his previous practice.

This thwarted expectation resulted in a temporary breakdown in his routine performance of the new practice and subsequent contextual sensemaking to further optimize the practice of sharing work materials online via the possibilities offered by the screen-sharing function of a video conferencing platform: *“With Microsoft Teams, for example, … just being able to make a call, be able talk and show what’s on your screen at the same time. We would be lost without it”* (Liam). When performed, this adjusted sociomaterial practice more authentically mimicked the activity of physically looking at the same computer monitor in the organizational office. Even using one’s finger to point out certain aspects on the monitor was able to be replaced with cursor movements on the shared screen.

One of the biggest challenges that our participants encountered when being forced to work from home, was an awareness of others’, as well as signaling their own, availability for ad-hoc communication: “*All of a sudden, you’re locked in this virtual world where your colleagues might be in another meeting, but you’re not going to be able to disturb them because they’re in another world”* (Jacob). Working as dispersed individuals in their homes, they lacked the sense of co-presence that physical co-location previously gave them:

We don’t know what's really happening around us. We don’t know how busy people are. I feel bad randomly ringing people out of the blue ... Sometimes when I’m really busy and someone calls me three times a day … it’s hard to manage. Whereas, at the office, they’d be seeing, ‘She’s busy. I’ll talk to her about this later.’ (Rajani)

This sometimes had implications for knowledge sharing and work productivity as previously ad-hoc in-person communication became no longer possible: *“I think that people are struggling with stuff and can’t easily ask for help. You can’t just say, ‘Hey, can you take a look at this?’, or, ‘Hey, I’ve got a question’”* (Rachel). On the other hand, for some participants, the shift to a non co-located actor configuration in working from home offered opportunities for undisturbed work compared to the organizational office where they could be easily interrupted through physical encounters with their colleagues. In the following example, Pete recounts how his sensemaking around the sociomaterial practice of online ad-hoc communication enabled him to reduce his accessibility by taking advantage of the possibilities offered by the reliance on a messaging program to enact that communication:

I’m head of our office, so people just randomly will walk up and ask me stuff, and I can’t just ignore them at work. Here, if someone Slacks me, I don’t have to answer. It’s easy to actually stay focused without having to stop and address something that’s come up. (Pete)

To overcome the disruption to their practice represented by the absence of visual cues in signaling one’s availability, many of our participants used digitally mediated notifications to increase workplace awareness. As Wendy explained:

I’ve been using status updates more … Where people might see you walk by in the office or they might see you down in the lunchroom … here [working from home], they don’t have that … You have to find new ways to communicate when people don’t get the non-verbal clues that they used to. (Wendy)

Similarly, by using status updates offered by an online messaging program, such as “*offline, online, lunch*”, Oliver was able to signal his availability to colleagues: *“Your Slack status has to symbolize what you are [up to], or if you’re in a meeting, for example.”* Such measures allowed colleagues to respond attentionally to these status updates, reducing uncertainty and conditioning them to work on other tasks. Of course, the possibility of constant digital connectivity could itself form an interruption to the practice of work that required managing from within that sociomaterial practice, as Salim explains:

I don’t want to have numbers of notifications in the corner of my applications … When I have a notification in Teams, I love to read and reply which is very disruptive … What I do to manage it, … I turn the notifications off … Otherwise, it’s really interruptive. (Salim)

### Online Socializing

Socializing with colleagues at work was perceived as an important mechanism for establishing and maintaining the social connections that enhance employee well-being and working in teams. For example, Alexander lamented the missed opportunities for social interaction with his colleagues: *“Talking a lot of just rubbish all day … It’d still be nice to have some office time again, just to talk to everybody.”* Similarly, Nick was concerned about *“the engagement aspect of feeling like a team and feeling the sense of collective purpose.”* The absence of what was previously taken for granted caused many of our participants to consider how social connections could be maintained in a dispersed online environment.

Daily check-in meetings became seen as obvious and legitimate ways to renew the practice of maintaining social ties with co-workers, ensuring each other’s well-being, supporting members of a team, and providing the opportunity to discuss both private and professional concerns. For Anna, this was clearly an essential part of her experience of work that needed to continue after lockdown:

The reason we started having meetings every morning was so that we’re still connected, and everyone feels like there’s someone they can talk to. And we can keep in contact with knowing what’s going on both in our personal lives and working lives ... It helps us feel connected and the days that, for example, if you don’t have meetings, I feel like I’m not even at work. (Anna)

While in-office socializing often occurred spontaneously during the working day in the form of ‘water-cooler’ chats between small numbers of colleagues, the virtual check-in meetings were often planned and held at specific times of the day with the entire team to bring everyone together in the same virtual space at the same time: *“We do a daily check-in at 8:45 in the morning … just to get a gauge of how everyone’s feeling that day”* (Garry).

Similarly, the impossibility of holding in-person traditional social events such as after-work drinks, whether in the office or utilizing the facilities of nearby pubs or bars, left a gap in the social calendar of many participants that they attempted to fill using a range of regular (often weekly) online social events such as virtual drinks, quizzes, or themed dress-up events. The primary purpose of these events was to maintain office traditions, talk about non-work-related topics, and combat isolation, especially for those colleagues living by themselves: *We’ve got a bubble quiz later on this afternoon [laughs]. We’re doing some social stuff like that. You’re at home by yourself or you bring your family in and have a drink and answer some questions or do some fun things like that”* (Nick). Similarly, Garry explained, *“We’ve got a Harry Potter day tomorrow, so they try and find a wizard hat or something … It’s a bit of fun … [to] get people laughing a little bit, which I think is really important at a time like this, where people might be feeling isolated”*.

Some of our participants experienced temporary breakdowns in their performance of these renewed practices of online socializing that required deliberate sensemaking while remaining within the practice to repair the disruption. For example, Matt became uncomfortable with the idea of using work-provided online communication platforms for social events with colleagues: *“Who knows if this has been captured or stored somewhere, right? Sometimes the social drinks, maybe some of the content we wouldn’t say publicly if we were in a work environment, if you know what I mean.”* His discomfort focused his attention on the potential for the digital technology used to become a management tool for monitoring staff behavior. Making sense of how the technology was enacted in the online socializing practice suggested the use of other, more independent platforms for these social events.

Other participants experienced thwarted expectations arising from the limitations of the virtual environment compared to the in-person social events before lockdown: *“We had a better conversation than we do now on Zoom … It’s weird because you have to wait for the other person to finish. In the office, … we’d have our little mini-groups, and we switched around … Here it’s one conversation in a way”* (Rajani). In response to this sort of temporal breakdown, other participants began avoiding the larger organizational events in favor of enacting online socializing with a smaller actor configuration where they could talk more freely and engage in more personal conversations: *“Last Friday, for example, we didn’t go to the company [event] but we made our own call because then we could make our inside jokes. We could talk about more team internal stuff or like because we know better the families of the people in the team”* (Oliver).

### Managing Work Space and Time

With the move to enforced working from home, the geographical and temporal boundaries between work and private life were suddenly disrupted: *“What happens is then that, really, you don’t have the time separation, and you don’t have the space separation either. It’s really blurry now”* (Patricia). For many individuals, work and private life were now performed in the same constrained space, with virtually present colleagues and clients juxtaposed with private actors such as partners and children. Managing their work space and time became essential to keep some form of separation between these two worlds.

Work extension became a significant problem for some participants as without an established end of work time as in the organizational office environment, or a physical commute to separate work from non-work, there was a tendency to continue working until later because the sociomaterial arrangement of working at home was ‘ready at hand’: *“Here the computer is there all the time, so it’s easy to say, okay, I’ll just do this task”* (Mansour). Confronted with the disruption represented by overworking, participants focused on the possibilities offered by specific material aspects of their environment to make various adjustments to their working from home practice that would overcome the perceived problem.

One example included setting a separate alarm to signal the end of work time: *“I literally put a physical alarm, the alarm that doesn’t have any Internet … The alarm will start to be like, ‘Ding, ding, ding’ … Yes, to remind me, ‘Okay, whatever I'm doing now, I need to log off’”* (Sandra). Those with caring commitments for others, including their children, often had to arrange their work time differently from the typical working day. Here, Hamza, a parent, describes making adjustments to the timing of her work and private commitments in order to accommodate the demands of both worlds:

I usually work until almost 3 or 4 am in the morning. I know it is killing me, I have headaches … I'm writing at this time [in my PhD] so I need to think a lot … I need a peaceful environment and that’s not possible in the daytime when my son is up. (Hamza)

A common issue confronted by our participants was making material arrangements to construct a suitable work space without necessarily the features they were used to in the office setting, such as ergonomic furniture. People improvised, sensing affordances in different facilities and artifacts available in their private homes. Examples include putting their laptops on an ironing board to serve as standing desk, working at the communal dinner table, or repurposing a spare room as a working from home office. This latter configuration utilized the physical layout of their home space to maintain a separation between work and non-work: *“When I come to this room, this means my office. When I go to that room, it means home”* (Salim). Here, the allocation of a work space physically separate from where private life took place was an actionable meaning within his practice of working from home that Salim derived from his deliberate sensemaking on this issue. Others were more constrained in their available spaces and needed to use their bedroom for work, which was at least separated from their main living area, as Sandra explained:

I realize it’s not helpful for my mental [well-being] because if I work in the living room, my work and my life are just one foot away. It certainly doesn’t help me to relax. I just keep thinking about work and I start to feel a bit stressed … Now I’ve moved it [work] back here [the bedroom] because here it is just work … [or] sleeping time. (Sandra)

One of the most common disruptions to their practice of working from home described by our participants involved ‘boundary crossings’, where an aspect of private life was perceived to intrude into work activities. Examples included other actors in the home and physical objects like a mounting laundry basket, dirty dishes in the kitchen, or a playing television: *“Sometimes [I’m] trying to concentrate while the kids are upset, crying, or have been fighting … [chuckles]. It can be really challenging. Or if you’ve got a meeting and one of them bursts into the room and they’re crying. You feel embarrassed, but what are you going to do?”* (Liz). Responses to such temporary breakdowns in their absorbed coping included the use of virtual backgrounds or the mute function in video conferencing technologies to remove the source of the disruption or the use of visual cues to signal appropriate behavior from family members and flatmates.

Hamza explained how she resolved the tension between her busy home life and her cultural expectation of presenting a tidy home to visitors by blurring her background in online work meetings: *“Everyone could see the mess behind my head [laughs] … I don’t want to show it. It was a comfort to know that I can blind something behind me … I really don’t have time to clean my table behind me.”* In contrast, Laura deliberately did not attempt to disguise the co-location of her work space with artifacts from her private life. Her sensemaking around this issue rendered the boundary crossing between private life and work as an expression of her personality and personal circumstances:

I could have hidden everything behind me, but I don’t care … I’ve clearly got another little desk and that’s where my daughter is working. I've got a Lego model ... You can see there’s a mess behind me … I have no issue with this because it's how I am and it’s what makes me, me. (Laura)

As well as visual intrusions, sound often crossed the boundary between private life and work and disrupted the performance of working from home. Rajani explained how she avoids her family members disrupting her work meetings by leveraging the mute functionality of the video conferencing platform she uses:

Usually when I’m not talking, I put myself on mute, because everyone in my household screams when they talk. Every time I'm getting up [to speak in the meeting] I’m like, ‘Okay, everyone shut up. I need to talk for five minutes and then after that, you can just carry on. (Rajani)

Sometimes the disruption is to other participants in the virtual meeting, and they need to intervene to restore the smooth flow of the online meeting practice. Patricia described one such incident where her daughter came into her home office to show her a piece of art she had made at school. Patricia began praising her daughter without realizing that the unmuted audio functionality of the video conferencing platform she was using was broadcasting her private conversation to her work colleagues: *“[I said,] ‘Look, it’s fantastic … you’ve got a real skillset that’s going to be fantastic for the future,’ then somebody said, ‘Just to let you know, you're not on mute.’”.*

Communicating appropriate behavior to the other household members could be significant in avoiding intrusions from the private sphere into our participants’ work activities. For example, Laura explained how she manipulates the materiality of the door to her home office to signal to her children whether they are allowed to interrupt her while she is working or not. In this case, a closed door is given meaning and is positioned in her sociomaterial practice as enacting a boundary between her private and professional worlds:

If the door is closed, don’t [come in]. I keep my door open the majority of the time. The only time I shut my door is if I’m having a very sensitive conversation … It’s not so much about me, it’s about the person on the receiving end [of the call]. (Laura)

### Accessing Organizational Resources

Prior to the pandemic-related lockdowns, our participants had ready access to their organizational offices and the physical and digital resources that they usually drew on in their work practice, including network connections, hardware and software, databases, peripheral devices such as printers, and various artifacts that they worked on. The sudden move to enforced working from home sometimes placed unexpected constraints on their access to these resources. These included access to high-bandwidth broadband, dedicated servers and computers, and the availability of organizationally provided technical support. Instead, in abruptly becoming remote workers, some employees ended up reliant on home Internet and Wi-Fi connections, personal devices, and whatever resources they had managed to retrieve from their offices before the lockdown. This could sometimes lead to technically-generated disruptions in their working from home that necessitated accommodations to how work was performed: *“I’ve had a bit of a dodgy Internet connection though, so that hasn’t helped. Also, my webcam isn’t working on my laptop, so I can’t see my face, but we can talk. It’s fine”* (Liz).

Without the appropriate hardware, software, and networking components that enabled them to perform their work tasks as before, executing specialized tasks could also be problematic. Mansour was challenged to rethink his practice of retrieving information from an organizational database. Working remotely, his attempt to do so would adversely impact organizational system processes: *“To create the report, I cannot connect to the production systems and just run some queries, because that will slow down the day-to-day activities.”* His solution involved enlisting the assistance of a colleague who had access to the organization’s VPN and could transfer data to an additional server. This resulted in faster execution of database queries without slowing down other systems:

The first part was to export all of the data from production machines onto … a staging server where we can run these queries. Getting that in place was big challenge. It took about three or four days for him to get the data off the production service on this secured staging area. (Mansour).

This reconfiguration of Mansour’s practice involved enrolling another remote working colleague, who became enacted as an additional social actor in the reshaped practice, and being attentive to the possibilities offered by the digital infrastructure of the organization.

Similarly, to complete one of his daily work routines, Pete needed to access a database on a dedicated local office computer. Remote access to the dedicated machine while working from home was problematic and involved paying deliberate attention to what was available in terms of technical possibilities and finding a solution that enabled the activity to continue, particularly with the increased demand on the organization’s digital infrastructure from most employees working from home: *“We’ve had to create some interesting workarounds to be able to remotely access that computer, to get access to those applications … We also used it to test the capacity of our VPN, which we also discovered did not have enough capacity, so we switched VPN provider”* (Pete).

## Discussion

The goal of this research study was to answer the research question: *How do employees navigate enforced working from home and how do their sociomaterial practices of work change as a consequence?* Given the sudden and significant disruption represented by the COVID-19 pandemic and its associated lockdowns, we wanted to theorize the changes that occurred when the locus of work shifted from the organizational office to the private space of a worker’s home. Taking a sociomaterial practice perspective allowed us to scrutinize the specific practice world (Sandberg and Tsoukas 2011,2020) of enforced working from home and to explore how employees became remote workers in an ongoing enactment of renewed and restored sociomaterial practices of work.

Our analysis identified five salient practices that required sociomaterial (re)configuration as workers confronted and made sense of the complete breakdown in their previously enacted work practice: scheduled online meetings, ad-hoc communication, online socializing, managing work space and time, and accessing organizational resources. Zooming in on the sociomaterial configuration of these five practices highlighted key differences in four generative elements of sociomaterial practice (Gaskin et al. 2014) available to actors in their work practices before and after the COVID-19 lockdowns, i.e., tool modality, actor configuration, actor location and nature of available infrastructure. These differences are summarized in Table 2.

**Table 2:** Generative elements of sociomaterial work practices

	Working from the organizational office	Enforced working from home
Tool modality	A blend of physical and digital tools such as the whiteboard in a meeting room (physical) or the project management software used to manage projects (digital)	Almost exclusively digital tools such as online digital whiteboards, video conferencing platforms, messaging applications
Actor configuration	Professional actors work individually or in groups	Professional actors work individually or in groups while engaging in activities with private social actors (e.g., home-schooling their children during what would be the normal working day)
Actor location	Professional actors often co-located with colleagues and clients in organizational offices Professional actors typically geographically separated from social actors in their private lives during working hours	Professional actors work from geographically distributed home environments, yet share the same virtual workspace Professional actors are co-located with private social actors in the home
Nature of available infrastructure	Physical office layout provides professional spaces such as offices, meeting rooms, kitchens Physical infrastructure includes ergonomic office furniture, computers, and peripherals Digital organizational infrastructure includes reliable broadband, locally stored databases, dedicated servers	Physical home layout provides private social spaces such as living room, bedrooms, home office Physical infrastructure may be limited to objects available in the home e.g., dining table, ironing boards, children’s desks Digital organizational infrastructure such as VPNs and cloud-based databases may be available Digital private infrastructure includes personal Internet connection, home computers, mobile phones

Tool modality was an important constitutive component of the enacted practices. While work before the lockdown incorporated both physical and digital tools, the pandemic dramatically increased reliance on the use of digital technologies as tools in the reconfigured work practices when working from home. For example, while actors previously had access to paper-based files, physical whiteboards, and computer monitors in their organizational offices, working from home was enacted using predominantly digital files, video conferencing platforms, mobile phones, and laptop cameras. Enforced working from home also triggered a major shift in actor configuration and location. While employees used to work individually or in teams, often physically co-located in organizational premises, they now worked geographically dispersed in their individual homes. Despite their physical isolation, they continued to work and socialize collectively in a shared virtual space accessed through various digital platforms and communication channels. Nevertheless, the conjunction of work and private lives in the home created disruptions for both the workers and their colleagues, as well as for the household members they shared that private space with. Finally, the shift to enforced working from home had a significant impact on the available infrastructure to support work practices. Many workers did not have the separate space or ergonomic furniture needed to replicate a work office in their homes. Neither was the digital infrastructure routinely provided by their organizations always available, leading to a variety of local arrangements and workarounds in order to continue working.

For our participants, situated in the unfolding flow of their work practice, the complete breakdown in the routine enactment of their work represented by enforced working from home appeared extraordinarily problematic. Work as it was performed previously could, in many cases, no longer continue unchanged. As the relational whole of their prior practice broke apart, the disruption focused workers’ attention on the entangled social and material elements that previously constituted their practice when working in the office. Sandberg and Tsoukas (2011, 2020) argue that actors confront such situations by engaging in detached-deliberate sensemaking that attempts to review and understand key features of the newly problematized activities and conceptualize workable solutions that can renew routine practice. Our participants’ accounts provided evidence of their deliberate reconfiguring of the sociomaterial arrangement of work, which we suggest represents the intention to shape ongoing practice by exploiting the possibilities for action that they perceived in these (re)configurations.

However, those renewed sociomaterial practices were not themselves invariant in their enactment but became subject to further disruptions related to the withdrawing of one or more of the social or material generative elements constituting the practice in the flow of everyday actions. In general, such temporary breakdowns in absorbed coping (Sandberg and Tsoukas 2011) were highly contextual, shaped by individual professional and personal circumstances, the activity at hand, the actors themselves, and their perception of the disruptions to their practice. While remaining involved in the practice, actors were attuned and attentive to possible actions that could restore their interrupted practice. Again, our participants’ accounts provided indications of this ongoing adjustment and fine-tuning of practice (Mousavi Baygi et al. 2021) as they sensed affordances in the different social and material elements of their practice that momentarily came into view. Accordingly, what we observed in our data was not just employees’ immediate responses to the substantial disruption of their work practices under COVID-19 but evidence of the continued evolution of their renewed sociomaterial practices as they became tuned over time.

In responding to both complete and temporary breakdowns, the constitutive flow of our participants’ practice made specific sociomaterial configurations seem meaningful and ‘sensible’(Hultin et al. 2021). Situated in the flow of online meeting practice, it becomes obvious and legitimate to manipulate the camera or microphone, or virtual background to maintain a coherent practice. Similarly, within the practice of enforced working from home, one’s availability becomes enacted through the manipulation of status and notification functions in various online collaboration platforms. Our participants were also reciprocally shaped by their enactment of these renewed sociomaterial practices, becoming remote workers in particular and variable ways, even as they occupied other subject positions in this flow, such as the teachers of their children. For example, engaging in the sociomaterial practice of online socializing, they became enacted as part of a connected group of colleagues – a group that sees maintaining connections as an obvious and meaningful activity in the context of their working from home.

It is important to note that the interruptions to our participants’ absorbed coping in the flow of their practice were not exclusively disruptive. At times, the interruption was generative in the sense that the participant became aware of different possible practices through experimenting with the social and material elements of their sociomaterial practice. One example is the enactment of multi-channel communication during online meetings to minimize disturbances to the presenter by leveraging the chat function of the video conferencing platform being used. An enforced reliance on online whiteboards and project management applications led one participant to explore the added possibilities for visualization in online work presentations compared to in-person sessions. Finally, the implementation of online social events increased the opportunities for participation by team members who ordinarily worked non-regular hours when located in the organizational office.

### Theoretical Contributions

Our study makes three main theoretical contributions. First, we complement existing knowledge on enforced working from home (Caldeira et al. 2022; Carroll and Conboy 2020) by analyzing this empirical phenomenon from a sociomaterial practice perspective. Despite an increasing volume of research since the foundational work of Orlikowski (2007, 2010) and Suchman (2007), the term sociomaterial practice has tended to be primarily used to overcome the ontological distinction between the social and material by conceptualizing their constitutive sociomaterial entanglement. Holeman (2018, p. 289) argues that these “lofty conceptual debates lose sight of pragmatic concerns.” Thus, there is a dearth of studies that actually apply the theoretical lens to scrutinize activities and actions as part of organizational and work practices (Styhre 2011). We address this gap by generating a fine-grained empirical account that explores the specifics of how knowledge workers’ unfolding work practices changed under the initial COVID-19 lockdowns, with a particular focus on the collaborative and situated challenges that these workers faced and how they became remote workers by attentionally responding to the sociomaterial practices of enforced working from home that enacted them as such (Hultin et al. 2021).

Second, we operationalize Sandberg and Tsoukas’s (2011, 2020) theoretical framing of sensemaking in sociomaterial practices by locating the notions of complete and temporary breakdowns and subsequent conceptual and contextual sensemaking within the flow of practice perspective proposed by Hultin et al. (2021) and Mousavi Baygi et al. (2021). We use the synthesized theoretical framework presented in our paper to explain how workers’ responses to disruptions in their routine work practices represent different modes of sensemaking and how the underlying dynamics of their sociomaterial practices during enforced working from home are formed and adjusted in response to these, leading to change from within the conditioning flow of unfolding sociomaterial practices. In doing so, we illustrate empirically how this approach can help scholars explain when, how, and why sociomaterial practices change and continuously evolve in their embeddedness in the flow of everyday action.

Third, social science and organization studies have often downplayed the role of materiality in the development and performance of organizational routines and work practices (Feldman and Orlikowski 2011; Leonardi 2015; Styhre 2011). Given the importance and strong embeddedness of digital technologies in our everyday work practices (Gerson et al. 2008; Wajcman and Rose 2011), we contribute to the literature on sociomaterial practices by highlighting how specific digital technologies and tools become entangled in the change, renewal, and restoration of sociomaterial practices that are characteristic of enforced working from home. We foreground in our analysis not only the social actors but their relations with material objects that dynamically configure and reconfigure their practices and possibilities for action in working from the private space of their homes (Feldman and Orlikowski 2011).

### Practical Implications

**Fig. 2:**
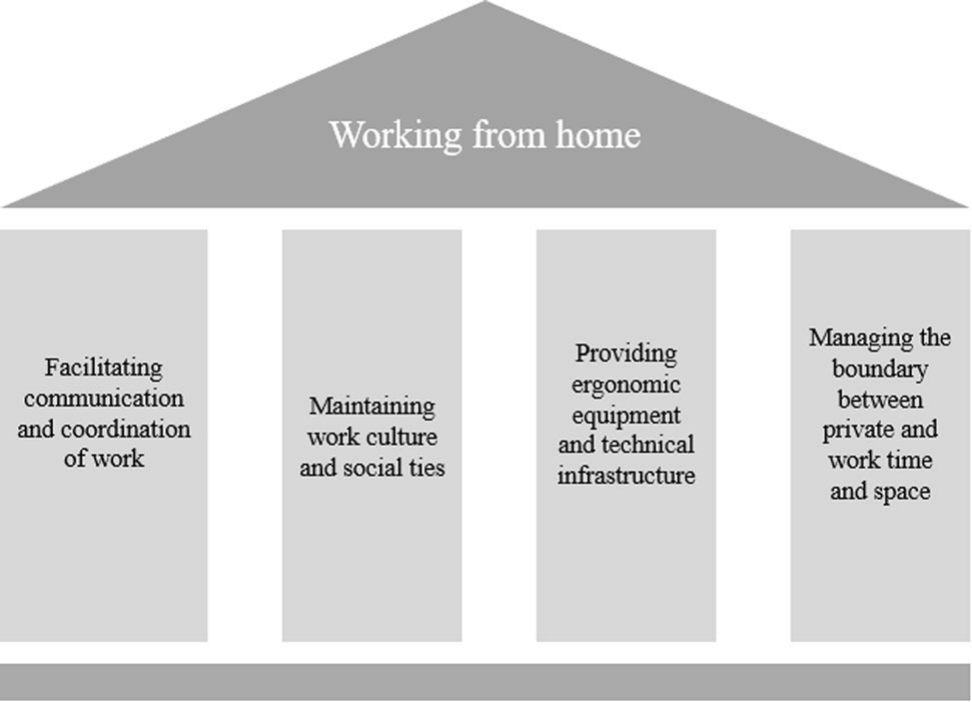
Supporting a conducive working from home environment

Our findings have practical implications that offer guidance for people working from home voluntarily or due to governmental ‘stay at home’ policies and for employers that are considering adopting hybrid arrangements for their workforce (Barrero et al. 2021; Office for National Statistics 2022). Figure 2 presents four pillars derived from the findings of our study that we suggest underpin and support the establishment of a conducive working from home environment: facilitating communication and the coordination of work, maintaining work culture and social ties, providing ergonomic equipment and technical infrastructure, and managing the boundary between private and work space and time.

#### Facilitating Communication and Coordination of Work

In order to achieve individual and collaborative work outcomes, regular communication among team members and the coordination and integration of interdependent tasks are fundamental. This relies on the collaborators jointly aligning their activities through an awareness of what is occurring in the work setting (Gutwin and Greenberg 2002; Kim et al. 2018; Schmidt 2002). Workers in flexible or non-co-located work settings often find it difficult to know if co-workers are available for consultation (Bardram and Hansen 2010; Bødker and Christiansen 2006; Kolfschoten et al. 2013). Because ad-hoc conversations are not scheduled in an online work environment, negotiating mutual availability can be problematic (Nardi et al. 2000). In our study, while focusing on their individual activities, workers engaged in a range of practices that monitored what their colleagues were doing and signaled their presence and availability for communication or collaboration. The materiality of different technologies and communication media provided different possibilities for doing this, including messaging programs and notification functions, and status updates on online collaboration platforms.

Video conferencing platforms have become the mainstay of online communication in working from home. Nevertheless, it is important to communicate expected behavior to minimize the effects of disruptions in online meetings, such as turning off the video functionality when a poor Internet connection is encountered (Saatçi et al. 2020). Examples that emerged from our findings included (a) switching on cameras to transmit non-verbal communication such as facial expressions and body language; (b) using virtual or blurred backgrounds if people feel uncomfortable sharing their private environment or need to maintain their professional identity, and (c) utilizing the in-built functionality of video conferencing platforms that facilitate multi-channel communication such as immediate reactions (e.g., thumbs up, clapping) or the chat function to avoid disrupting the main communication flow. While online meetings are an effective way to communicate among distributed workers, their overuse can lead to ‘Zoom fatigue’ (Nadler 2020) as well as “defeating the flexibility that makes working from home attractive” (Cappelli 2021, p. 69).

#### Maintaining Work Culture and Social Ties

Social ties to colleagues are crucial for employees’ engagement with their teams and organizations. However, building and maintaining social ties and a sense of connection between non-co-located workers often demands more effort from employers (Cappelli 2021). Hence, it is crucial to develop a strategy that facilitates the maintenance of the organizational culture and avoids feelings of isolation among remote workers (Ajzen and Taskin 2021). Examples of how this could be achieved derived from our study include regular online check-ins to discuss personal issues and check on each other’s well-being, virtual social events like online pub quizzes or themed online social functions, and engaging workers in challenges or competitions via enterprise social networks. However, it can be vital that the scale of online social events is kept relative to the objective. Having too many participants in an online event can lead to awkwardness and a lack of spontaneity or limit people’s ability to socialize. Further, the choice of platform used may need to consider the creation of psychologically safe online spaces.

#### Providing Ergonomic Equipment and Technical Infrastructure

Poor physical workspaces for working from home were commonly reported during the COVID-19 pandemic (McCarthy et al. 2020). Improvised arrangements, for example, working at the dining table or using an ironing board as a stand-up desk, can lead to physiological issues if used over the long term. As more and more employees seek to keep working from home (Barrero et al. 2021; Smite et al. 2022), organizations can provide education and training on the importance of an ergonomic workstation and could even follow the footsteps of big tech companies such as Google to support their employees financially in the establishment of an ergonomic workstation at home (Pichai 2020). Besides the physical set-up for work, employees working from home also require the necessary technical infrastructure, including hardware, software, and network connections, to maintain productivity and efficiency. These improvements can include additional screens, docking stations, cloud-based data management and storage, stable VPN connections, sufficient server capacity, and stable and secure Internet connections in the home office. Where remote access to organizational systems, databases, and servers is problematic, local arrangements dependent upon particular individuals and circumstances (Gerson et al. 2008) may emerge opportunistically and be challenging to support in the longer term.

#### Managing the Boundary Between Work Time and Space

Due to the possible drawbacks of the blurred boundaries between private and professional life (Pedersen and Lewis 2012), employees working from home can develop and use a range of strategies to maintain their work productivity and focus, while protecting their private lives from work-related interruptions. Those strategies may include (a) physically separating work from private life by using a dedicated space such as a guest room, the garage, or another separate space as a designated work area; (b) establishing norms and rules for household members to reduce interruptions when engaged in critical or sensitive work; (c) setting up regular work hours that are compatible with private life demands (e.g., child care); (d) using pre-set cues to signal the end of work time, and (e) using different devices such as work and private phones to separate the two spheres.

### Limitations and Avenues for Future Research

Our research has two limitations that need to be acknowledged, but also presents promising opportunities for future research. Our analysis of how sociomaterial work practices changed during enforced working from home is based on retrospective interview accounts provided by our participants at the beginning of the pandemic in 2020. Despite the relatively short timeframe of their navigating working from home, we could observe that after the initial complete breakdown and subsequent renewal of their work practices, our participants made further adjustments to the sociomaterial configuration constituting their practice in response to ongoing temporary breakdowns as they became accustomed to working in these new ways (Sandberg and Tsoukas 2011, 2020). Nevertheless, it would be interesting to understand how this evolution of sociomaterial practice unfolds in the long term when actors become habituated to working from home or adopt hybrid working (Barrero et al. 2021; Office for National Statistics 2022; Smite et al. 2022). Further research is needed that adopts a longitudinal approach to theorize when, how, and why those sociomaterial practices evolve in other work models than enforced working from home (Waizenegger et al. 2020).

While temporary and complete breakdowns and their associated kinds of sensemaking suggested by (Sandberg and Tsoukas 2020) served as valuable theoretical concepts to help us explain the renewal and adjustment of sociomaterial practices in the context of enforced working from home (Mattern et al. 2021; Waizenegger et al. 2020), our data did not allow us to examine the specific sensemaking of each participant as it occurred in practice. This constitutes an exciting opportunity for future research to empirically unpack the black box of the sensemaking process with regard to work practices in the home office and also the role of the situational factors in influencing the actual process of sensemaking in this context (Sandberg and Tsoukas 2015). Our research suggested that people’s individual context, in particular their living situation, their professional and private identity, the technology that they adopted and used, and their emotions, influenced their sensemaking process. Further research is needed to explore how these situational factors become relevant and achieve their effects.

## Conclusion

Based on insights from 29 interviews as part of our qualitative research study, we examined the particular sociomaterial practices of working from home during a pandemic and identified five emerging practices associated with enforced working from home (Mattern et al. 2021; Waizenegger et al. 2020). These were scheduled online meetings, ad-hoc communication, online socializing, managing work space and time, and accessing organizational resources. In doing so, we contributed to addressing the lack of understanding of digital technology-driven work practices that have become important in this context (Carroll and Conboy 2020; Tønnessen et al. 2021). Improving our understanding of the challenges people face while working from the private space of their homes and the evolving nature of their sociomaterial practices contributes to the academic discourse on working from home. It may also help organizations and employees shift from the emergency state of enforced working from home to embracing the ‘new normal’ of work (Carroll and Conboy 2020; Franken et al. 2021).
